# Association of the CpG Methylation Pattern of the Proximal Insulin Gene Promoter with Type 1 Diabetes

**DOI:** 10.1371/journal.pone.0036278

**Published:** 2012-05-02

**Authors:** Delphine Fradin, Sophie Le Fur, Clémence Mille, Nadia Naoui, Chris Groves, Diana Zelenika, Mark I. McCarthy, Mark Lathrop, Pierre Bougnères

**Affiliations:** 1 Institut National de la Santé et de la Recherche Médicale U986 and Department of Pediatric Endocrinology, Bicêtre Hospital, Paris Sud University, Paris, France; 2 ISIS-DIAB Cohort, Bicêtre and Saint Vincent de Paul Hospital, Paris, France; 3 Centre National de Génotypage, Evry, France; 4 Oxford Centre for Diabetes, Endocrinology and Metabolism, Churchill Hospital, Headington, Oxford, United Kingdom; University of British Columbia, Canada

## Abstract

The insulin (*INS*) region is the second most important locus associated with Type 1 Diabetes (T1D). The study of the DNA methylation pattern of the 7 CpGs proximal to the TSS in the *INS* gene promoter revealed that T1D patients have a lower level of methylation of CpG -19, -135 and -234 (p = 2.10^−16^) and a higher methylation of CpG -180 than controls, while methylation was comparable for CpG -69, -102, -206. The magnitude of the hypomethylation relative to a control population was 8–15% of the corresponding levels in controls and was correlated in CpGs -19 and -135 (r = 0.77) and CpG -135 and -234 (r = 0.65). 70/485 (14%) of T1D patients had a simultaneous decrease in methylation of CpG -19, -135, -234 versus none in 317 controls. CpG methylation did not correlate with glycated hemoglobin or with T1D duration. The methylation of CpG -69, -102, -180, -206, but not CpG -19, -135, -234 was strongly influenced by the cis-genotype at rs689, a SNP known to show a strong association with T1D. We hypothesize that part of this genetic association could in fact be mediated at the statistical and functional level by the underlying changes in neighboring CpG methylation. Our observation of a CpG-specific, locus-specific methylation pattern, although it can provide an epigenetic biomarker of a multifactorial disease, does not indicate whether the reported epigenetic pattern preexists or follows the establishment of T1D. To explore the effect of chronic hyperglycemia on CpG methylation, we studied non obese patients with type 2 diabetes (T2D) who were found to have decreased CpG-19 methylation versus age-matched controls, similar to T1D (p = 2.10^−6^) but increased CpG-234 methylation (p = 5.10^−8^), the opposite of T1D. The causality and natural history of the different epigenetic changes associated with T1D or T2D remain to be determined.

## Introduction

Type 1 diabetes (T1D) results from the autoimmune destruction of insulin-producing beta-cells caused by yet unknown environmental factors in genetically predisposed individuals. While the complexity of T1D causality is yet to be unraveled at the biological and epidemiological levels [1], epigenetics can be suspected to contribute to T1D causality as a reflect of environment-gene interactions.

The current study focuses on the insulin (*INS*) locus. The genetic polymorphism of DNA sequence at the *INS* locus has been associated with T1D as the first application of the TDT (Transmission Disequilibrium Test) approach to multifactorial disease genetics [2]. Since then, it has been consistently confirmed that T1D is associated [3], [4], [5], [6] with the A/T SNP called rs689 and located at position +215 bp of the *INS* gene TSS. The MHC locus being IDDM1, the *INS* locus was named IDDM2, because it has the second highest odds ratio (OR) for T1D risk [7]. In Caucasians, rs689 is in complete linkage disequilibrium (LD) with the VNTR classes, allele A being associated with short class I VNTR alleles, more frequent in T1D, while allele T is associated with long class III alleles. Studies of *INS* gene expression suggested that while given class I alleles are associated with increased *INS* expression in a rodent ß cell line [8] and human pancreas [9] versus class III alleles, the opposite observation was obtained in whole thymic tissue [9], [10]. Since rs689 did not seem to have *per se* a functional role in the regulation of *INS* gene transcription, it was proposed that sequence variation at the *INS* locus could affect *INS* gene transcription through a tissue-specific functional difference between VNTR classes. Possibly, epigenetic variation at the *INS* locus could provide an interface between the predisposing rs689 SNP and variation in *INS* gene transcription. This is because in mammals including humans, the DNA methylation of gene promoter regions is known to play a role in the transcriptional regulation of many genes, most often in a tissue specific manner [11]. This was shown specifically for the *INS* locus where CpG methylation of the promoter was able to modify gene expression in β cell lines and islets of T2D patients [12], [13]. The variation of DNA methylation within the *INS* gene promoter, a low-CpG containing region, may thus be suspected to regulate *INS* gene transcription in pancreatic ß cells and medullary thymic epithelial cells (mTEC), the two tissues that express this gene and are central to the mechanisms of T1D [14]. Since these tissues cannot be studied in T1D patients, we undertook the study of DNA methylation at the *INS* locus in whole blood cells (WBC) as a possible surrogate of epigenetic marks in INS expressing tissues [15].

To explore if chronic hyperglycemia could be suspected to cause the observed changes in the methylation pattern of the *INS* promoter, we studied a group of T2D patients compared with age-matched controls.

## Results

Using a bisulfite-PCR method [16], we measured DNA methylation of 7 CpGs located in the proximal part of the *INS* promoter in 485 T1D patients compared with 317 age-matched non diabetic individuals (Table 1).

**Table 1:** Main characteristics of T1D patients and age-matched non diabetic controls.

	T1D Patients	T1D Controls
**N**	*485*	*317*
**Sex (M/F)**	259/226	163/153
**Current age (yrs)**	12.1±4.9	11.8±4.8
**BMI (kg/m^2^)**	18.4±3.2	23.3±10.6
**Age at clinical onset (yrs)**	6.3±3.1	-
**Diabetes Duration**	7.5±8.5	-
**Hba1c**	8.1±1.4	-

Results are expressed as mean ± sd.

### Association of CpG methylation at the INS locus with T1D

**Figure 1:**
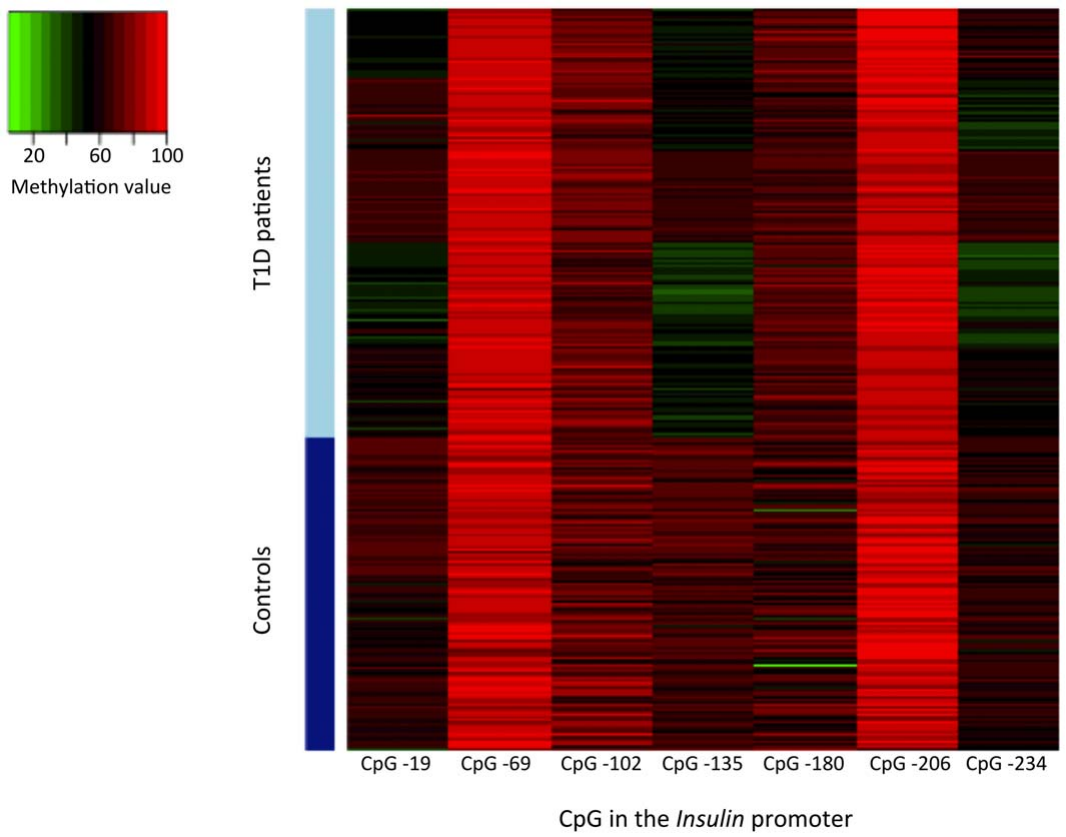
Heatmap analysis of the relationship between DNA methylation of the whole proximal *INS* promoter and disease status. Each row represents one individual.

**Figure 2:**
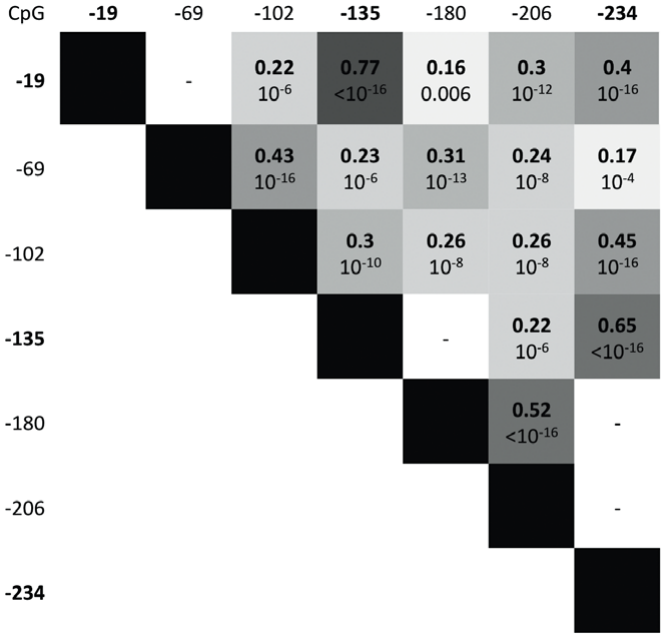
Correlation matrix of the methylation values (%) at the *INS* promoter CpG sites in T1D patients (R in bold, p-value below). The T1D-associated hypomethylated CpGs -19, -135 and -234 are highly correlated (<10^−16^).

The CpG methylation pattern of the proximal part of the *INS* gene promoter showed distinctive characteristics in the T1D patients. First, there was no T1D-related global directional change in methylation level that would affect all CpGs equally, nor did neighbouring CpGs adopt systematically the same changes in methylation. Compared with controls, CpGs -19, −135 and −234 in T1D patients showed a decrease in methylation (ranging from −10 to −16% of the mean, p<2.10^−16^), while methylation of other CpGs was either unchanged (CpGs -69, −102, −206) or slightly increased (CpG -180, +3% of the mean, p = 1.10^−6^) (Table 2, Figure 1). Defining relative hypomethylation of a given CpG as a value below −2SD of the corresponding control level, we found that 70/485 (14%) T1D patients had a simultaneous hypomethylation of CpG -19, -135 and -234, while 0/317 controls showed such triple hypomethylation (p<2.10^−16^). Methylation levels of the four T1D-associated CpGs, although not in immediate vicinity on the DNA phase, were closely correlated (0.40<r<0.77, p<2.10^−16^, Figure 2), suggesting a shared process of regulation of the methylation status of these CpG residues, that is also seen in controls. Methylation levels of other CpGs showed a weaker degree of correlation between them as well as with the T1D associated CpGs (Figure 2).

**Table 2:** CpG methylation in the *INS* promoter in T1D patients and controls.

	T1D Patients (*N = 485)*	CONTROLS *(N = 317)*		T1D Patients *(n = 485)*	CONTROLS *(n = 317)*	
	DNA methylation (%)		*p*	Hypomethylated subjects N (%)		*p*
**CpG -19**	54±10	64±6	<2.10^-16^	190 (39%)	12 (4%)	<2.10^−16^
**CpG -69**	90±4	89±6	0.65			
**CpG -102**	73±6	73±9	0.11			
**CpG -135**	52±9	68±5	<2.10^−16^	273 (56%)	12 (4%)	<2.10^−16^
**CpG -180**	69±7	66±11	1.10^−6^			
**CpG -206**	91±5	91±7	0.62			
**CpG -234**	54±9	62±6	<2.10^−16^	178 (37%)	8 (2%)	<2.10^−16^
**CpGs -19, -135, -180**	53±9	64±6	<2.10^−16^	70 (14%)	0	<2.10^−16^

Results are expressed as mean ± sd. *P*-values are calculated by Wilcoxon rank sum test. Hypomethylated subject of a given CpG is defined by a methylation value below -2SD of the corresponding control level. We have only shown, for clarity, the hypomethylated subjects in the right part of the table. In contrast, note that methylation of CpG -180 is increased in T1D patients.

### Age-dependency and stability of DNA methylation in T1D patients

**Figure 3:**
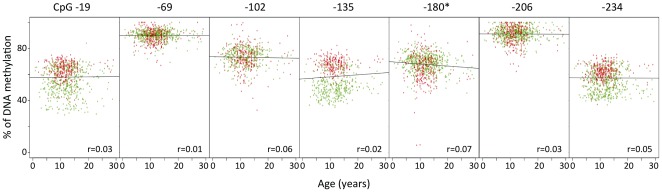
Lack of correlation between age and *INS* promoter methylation in T1D patients (green) and Controls (red). Only CpG -180 methylation showed a slight downward trend with age (r = 0.09, p = 0.01). The figure also shows the distribution of individual methylation values, and the differences between T1D and control subjects for CpGs -19, -135, -180, and -234.

We found no correlation of methylation with T1D duration. We did not find either any trend of correlation with glycated haemoglobin (HbA1c), which reflects the degree of hyperglycemia to which patients WBC are chronically exposed (data not shown). We detected only a small trend of correlation of CpG -180 methylation with age (Figure 3). This may be due to the studied range of ages, which extends only from 1 to 30 years, but we know from analyses in older adult non diabetic subjects of another cohorts that there is a downward age-dependent trend of methylation for all CpGs located in *INS* promoter (Figure S1). T1D patients who were studied twice at 1 month to 5 years intervals, had consistent methylation values at all CpGs. Again, none of the studied CpGs showed any correlation with intra-individual changes in glycated hemoglobin values (Data not shown).

### Influence of rs689 genotype upon CG methylation at the INS locus

**Figure 4:**
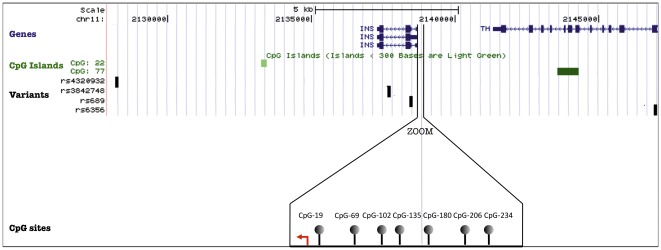
UCSC Genome Browser map of the *INS* locus : localization of genes, CpG islands (CGI), variants and CpG sites. Genes are represented by blue boxes (exons) and lines (intron). Under genes, CGI are in green. Green intensity and size of the box is function of the size and the number of CpG included in CGI. Polymorphisms are represented by black boxes, under CGI. Below, a zoom of the *INS* promoter allowing the localization and representation of the isolated CpG sites, according to the TSS, represented by the red arrow.

**Figure 5:**
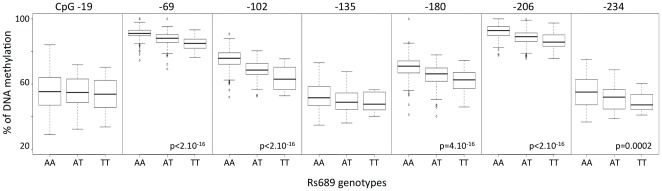
Boxplot of methylation values of the studied CpGs in the *INS* promoter with respect to the rs689 genotype in T1D patients. Except for the CpG -180, the T1D-associated CpGs show no direct association with the genotype. P-values are calculated by Kruskal-Wallis test.

**Figure 6:**
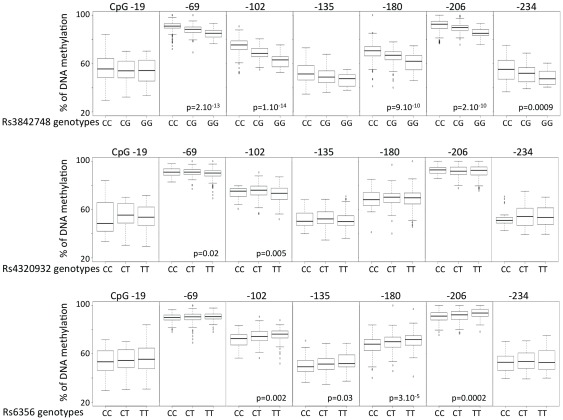
Boxplot of methylation values for the 7 CpGs in *INS* promoter with respect to SNPs other than rs689 in T1D patients. Methylation at CpGs -69, −102, −180 and −206 are associated with rs3842748. More distant SNPs showed a much weaker correlation with methylation at CpG -69 and -102 for rs4320932 and with methylation at CpGs -102, -135, -180 and −206 for rs6356. See Figure S1 for SNP locations. P-values are calculated by Krukal-Wallis test.

We found a strong and consistent correlation between the methylation status of 4/7 CpGs and the rs689 genotype (Figures 4 and 5), indicating that the T1D-predisposing A allele is consistently associated with higher methylation levels of CpGs -69, -102, -180, -206 (Figure 5). Note that CpG -180 hypermethylation is associated with T1D. Not all rs689 neighboring CpGs showed a dependency on this SNP, since rs689 had very little influence on methylation values of CpGs -19, −135, −234, the three other CpGs whose hypomethylation was found to be strongly associated with T1D. A comparable genotype-epigenotype relationship was also observed for rs3842748, a SNP in high linkage disequilibrium with VNTR classes as rs689, but almost disappeared for the more distant SNPs rs4320932 and rs6356 located on either side of the *INS* locus (Figures 4 and 6). The distribution of genotypes was not different in the patients having a decreased methylation of the 3 CpG -19, -135, -234.

### Association of CpG methylation at the INS locus with T2D

Compared with age-matched controls, T2D patients showed a decrease in methylation at CpG -19, and an increase at CpG -234, while methylation of other CpGs showed no or marginal (CpG-102) changes (Table 3). There was no correlation between CpG-19 and CpG-234 changes in the T2D patients. Neither CpG-19 decrease nor CpG-234 increase in methylation were related to T2D duration. None of the T2D patients showed a simultaneous hypomethylation at CpG -19, -135, and -234, the distinctive pattern observed in T1D patients.

**Table 3:** CpG methylation in the *INS* promoter in T2D patients and controls.

	T2D Patients *(N = 132)*	T2D CONTROLS *(N = 186)*	
	DNA methylation (%)		*p*
**CpG -19**	47±11	54±10	2.10^−6^
**CpG -69**	88±11	88±6	0.22
**CpG -102**	68±18	64±13	0.01
**CpG -135**	58±10	55±10	0.06
**CpG -180**	57±19	61±11	0.14
**CpG -206**	91±10	93±7	0.79
**CpG -234**	57±9	51±6	5.10^−8^

Results are expressed as mean ± sd. *P*-values are calculated by Wilcoxon rank sum test.

## Discussion

The current study investigated the DNA methylation pattern of the *INS* promoter because the common genetic variation at rs689 is known to be strongly associated to T1D, and because no clear functional role could be attributed to this SNP [2], [3], [4], [5], [6]. Among the 7 CpGs located within the proximal *INS* promoter, we found consistent methylation differences between T1D patients and non-diabetic controls at 4/7 CpGs. These 4 CpGs were located −19, −135, −180 and −234 bp from the TSS. Methylation at the other 3 neighboring CpGs showed no difference between T1D patients and age-matched controls. The small magnitude of the differences observed at CpGs -19, -135, -180, -234 is consistent with those reported recently by Rakyan et al in T1D twins studied with a methylation array [17]. By combining the changes in methylation at CpG -19, -135, and -234, it becomes possible to define a 3-CpG-hypomethylation pattern that seems to be present only in T1D patients, thus could be specific and predictive of T1D, if confirmed by follow-up studies. This 3-CpG pattern is present in only 14% of T1D patients, thus is not sensitive as a biomarker.

We found surprising that T1D was associated with a gain of methylation in one CpG (−180) and with a loss of methylation in 3 others, and we have no explanation for this observation. From a general point of view, changes in DNA methylation, whether it is an increase or a decrease for *cis-l*ocated CpGs, may be related to precisely located changes in the histone code and chromatin structure, since there are well established interactions between these epigenetic processes and DNA methylation [18]. If they occur commonly, such discrepancies between neighboring CpG of a given locus could be misleading in whole-methylome studies since the methylation of the CpG used to tag the locus may not reflect the methylation of other CpGs located at other positions over the whole locus

Another question encountered by epigenetic epidemiology is whether the observed methylation changes pre-exist or follow the establishment of the disease state. Since this cannot be inferred from the current data, interpretation of the T1D associated changes in DNA methylation should remain open to both hypotheses. We are however encouraged to postulate that the observed epigenetic pattern may contribute to T1D predisposition or autoimmune mechanisms by the recent pioneering study of Rakyan et al., who identified 132 T1D–associated methylation variable positions in 15 T1D discordant MZ twin pairs [17]. This latter approach did not detect the epigenetic pattern that we found associated with T1D at the *INS* locus. This apparent discordance can be explained by the methods used by these authors. A difference is the greater statistical power of our case-control approach compared to their twin study. While we used a promoter-centered multi-CpG-per candidate region (7 CpGs within 250 bp) approach, Rakyan et al. used a genome wide one-CpG-per region approach. A following consequence of our observation for the epigenetic epidemiology of T1D, but more generally for the interpretation of epigenetic studies in multifactorial diseases, is that one CpG may not recapitulate the overall methylation changes in a given genomic region. This may explain why Rakyan et al. also using the CpG -180 as a unique marker of the proximal promoter region have not observed a significant association of this locus with T1D in the studied 15 twin pairs. When methylation arrays bearing for example 27,000 CpGs selected as markers among 28 millions CpGs on the human genome, are used for methylome-wide approaches, these arrays cannot encompass all CpGs of a given region. The high density DNA methylation arrays (Infinium HumanMethylation450 [19] and CHARM arrays [20]) and sequencing based technology, should improve the coverage of the methylome. However, as shown here, CpG residues in a given region may show variable degrees of association between their level of methylation and the disease, ranging from hypo- to hyper-methylation, and from nonsignificant to highly significant levels of statistical association. Thus it may be the heterogeneity of a methylation pattern that turns out to be characteristic of a disease. To further explore if exposure to chronic hyperglycemia could have resulted in the observed changes of CpG methylation, rather than the T1D disease itself, we studied a sample of non-obese patients with T2D that had a somewhat comparable duration of exposure to hyperglycemia. Comparison with age-matched controls revealed that T2D patients share the hypomethylation of CpG-19 with T1D patients, which could reflect a common consequence of the hyperglycemic status. However T2D patients showed no consistent trend for hypomethylation of CpG -135 or -234, a good marker of T1D. Instead, the level of methylation was increased at CpG-135 (non significantly) and at CpG-234 (p = 5.10^−8^). Again, the causality of such changes in T2D escapes the limits of the current report, and will be the matter of further investigation. Age differences could not explain the discordance between T1D and T2D patients. It is noteworthy that the two samples of controls also showed differences in their methylation patterns at the *INS* locus, as expected from an environmentally and age sensitive process. Further investigations in other groups of T1D, T2D patients, and age- and environmentally matched controls will be necessary to explain these differences.

The current observations raise unresolved questions regarding the functional meaning of the changes in the CpG methylation of the *INS* promoter found in the WBC of patients with T1D. For methylated CpGs to contribute to T1D mechanisms, methylation changes modulating *INS* expression should have occurred in ß cells and/or mTEC, according to our current view of T1D autoimmune pathogenesis. On the other side, if T1D pre-exist to the changes in methylation, such changes in CpG methylation may be caused by the metabolic perturbations related to the diabetic state. Since WBC are exposed to numerous changes in plasma composition in T1D patients, the DNA methylation at the *INS* locus could be affected through a dysregulation of the methylation machinery induced by T1D as an epigenetic signature of the diabetic state. If this is the case however, it would be difficult to understand the coexistence of increased and decreased CpG methylation associated with T1D at different CpGs since one would then expect the metabolic dysregulation to affect CpG methylation in a consistent direction. Our T1D patients were studied an average of 7.5 years after clinical diagnosis, a time when ß cell destruction is expected to be largely terminated. Thus we do not think that our observations in WBC interfere with those of Akirav et al., who found an increased proportion of demethylated DNA in the serum of 5 patients with recent-onset T1D, as a possible index of active ß cell death [21].

We found that CpG methylation was influenced by the rs689 alleles known to be in strong LD with VNTR classes in Europeans [22]. Genotype dependent variations of neighboring epigenotype have been reported for the FTO locus in WBC [23] and for several other loci across the genome (genome research) and called ASM (Allele Specific DNA Methylation, see review [24]). Shoemaker et al. observed ASM on 23% to 37% heterozygous SNPs in several cell lines [25] while Zhang et al. evaluated that ASM is likely to affect 10% of all human genes [26]. The finding of a link between genetic variants and epigenomic marks in T1D patients at the *INS* locus may provide a more general example of how genetic and epigenetic variation can be related. This may help our future understanding of the “missing heritability” enigma seen with many genome wide association studies of multifactorial traits or diseases, if it proves true that SNPs are only the markers that tag neighboring epigenomic variations. According to this view, certain SNPs showing strong statistical association with complex diseases may not have any functional effects *per se*, but may be associated with epitypes that have functional effects on gene expression.

The CpG -180 is the only one that is both associated to T1D and is influenced by the T1D-associated SNP rs689. The hypermethylation of this CpG methylation has a marked influence on *INS* gene transcription *in vitro* [12]. Indeed, Kuroda et al. showed that methylation of the CpG -180, located in a cAMP responsive element (CRE), suppressed *INS* promoter-driven reporter gene activity by 50%. In human pancreatic islets, Yang et al. also found that insulin mRNA expression correlated negatively with the degree of methylation of CpG -180 [13]. If such in vitro observations were to be true in the mTEC of T1D patients, this would support that CpG -180 has a functional contribution to the predisposing effect of rs689, a hypothesis consistent with the increased methylation of this CpG in T1D individuals carrying the A allele.

Decreased *INS* gene expression in the thymus of T1D patients has been suspected to drive the defect of tolerance leading to autoimmunity towards ß cells [9], [10].

Whether epigenetic changes pre-exist or are a consequence of T1D can only be established by long-term longitudinal studies of DNA methylation in subjects at risk for the disease. Since it will *a priori* remain almost impossible to investigate ß cells and mTEC in T1D patients, the question of tissue-specific methylation changes should have to be solved in animal models of T1D, like the NOD mouse. It is possible that the observed pattern of CpG methylation at the insulin locus may vary in other T1D and control populations as a reflect of gene-environment interactions proper to these populations. Until larger studies can be performed in such populations, the observed variations in DNA methylation should be considered restricted to the European people studied here.

## Materials and Methods

### Patients

Non-immortalized whole blood cell (WBC) samples were obtained from 520 participants randomly extracted from the Isis-Diab cohort (T1D cases) and from 317 controls. ISIS-Diab is an ongoing prospective cohort of T1D patients recruited by a French multicenter network composed of 77 diabetic centers (see list in Table S1) supported by the Programme Hospitalier de Recherche Clinique of the French Ministry of Health with the objective of studying gene-environment factors in young patients with T1D. To limit the risk of population stratification, all recruited patients are of Caucasian ancestry assessed by family history and grandparents' birthplace. Inclusion criteria are diabetes mellitus criteria, signs of insulin dependency, immediate insulin treatment and presence of GAD autoantibodies in serum. For the current study, we selected 317 Caucasian non diabetic controls to be sex and age-matched with the T1D cases.

A sample of 132 non obese T2D patients aged less than 65 years, studied < 7 yrs after T2D diagnosis, as well as 186 age- and BMI-matched controls, was obtained from UK to allow a comparison with the young T1D patients analyzed in the current study (Table S2).

Patients and controls were included in the study according to the French bioethics law with families being carefully informed and having signed a detailed informed consent agreed by CPP (number DC-2008-693; NI 2620, Comité de Protection des Personnes).

### Isolation of genomic DNA and bisulfite genomic conversion

Nucleic acids were extracted from WBC using the same phenol chloroform technique in French T1D and controls and using the same Promega DNA extraction kit in T2D patients and controls from UK. Genomic DNA was treated with EZ-96 DNA Methylation-Gold Kit, according to manufacturer's protocol (Zymo Research Corporation).

### Pyrosequencing

We PCR-amplified the bisulfite treated genomic DNA using unbiased nested primers (sequences on request) and performed quantitative pyrosequencing. Pyrosequencing was performed using a PyroMark Q96 ID Pyrosequencing instrument (Qiagen). Pyrosequencing assays were designed using MethPrimer (http://www.urogene.org/methprimer/index1.html). 200 ng of genomic DNA was treated with EZ DNA Methylation-Gold Kit, according manufacturer's protocol (Zymo Research Corporation) and amplified. Biotin-labeled single stranded amplicons were isolated according to protocol using the Qiagen Pyromark Q96 Work Station and underwent pyrosequencing with 0.5 µM primer. The percent methylation for each of the CpGs within the target sequence was calculated using PyroQ cpG Software (Qiagen).

### Genotyping

Genotypes were obtained from GWAS data (Illumina HumanHap300_(v1.0.0), HumanHap300v2_A, HumanCNV370v1_C, Human610-Quadv1_B). GWAS was conducted by the Centre National de Genotypage (CNG, Evry, France).

### Statistical analysis

Differences in DNA methylation of the insulin promoter between T1D or T2D patients and non-diabetic controls were analyzed using non-parametric Wilcoxon rank sum test. Correlations were calculated as adjusted R square that measures the proportion of the variation in the dependent variable accounted for by the explanatory variables. Methylation data were clustered using the heatmap.2 function of the R statistical computing environment. Differences in DNA methylation of the *INS* promoter according genotypes were analyzed using non-parametric Kruskal-Wallis test. Methylation analyses adjusted for age were conducted using logistic regression. Results are expressed as mean ± sd. “Relative hypomethylation” was defined as values of CpG methylation under -2SD of the distribution of values in age-matched controls of the same population of origin.

By default, the smallest p-value obtained by R is <2.10^−16^. All statistical analysis were conducted using R 2.10.1.

## Supporting Information

Figure S1 **Correlation between age and** ***INS*** **promoter methylation in two populations of controls, the young controls used in the current T1D study (red) and a cohort of older controls (black).**
(TIF)

Table S1 **List of the 77 diabetic centers by alphabetic order participating to the ISIS-DIAB network.**
(DOCX)

Table S2 **Main characteristics of T1D patients and age-matched non diabetic controls.** **Results** **are expressed as mean ± sd.**
(DOCX)
