# Supplementary figures and images for: Association of the CpG Methylation Pattern of the Proximal Insulin Gene Promoter with Type 1 Diabetes
Source: PLoS One. 2012 May 2;7(5):e36278. doi: 10.1371/journal.pone.0036278 (PMC3342174; doi:10.1371/journal.pone.0036278)

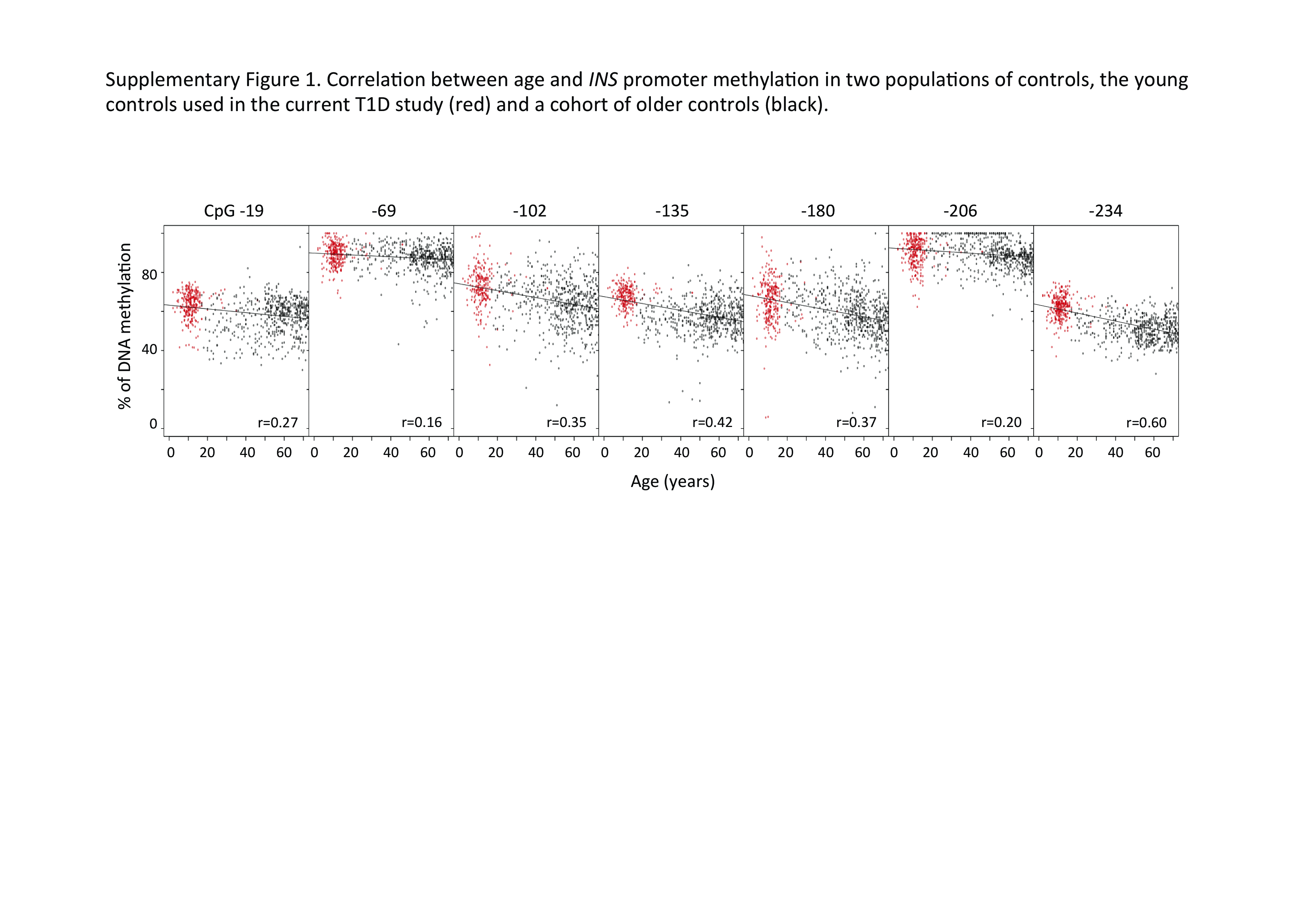

Supplement: Figure S1 — Correlation between age and INS promoter methylation in two populations of controls, the young controls used in the current T1D study (red) and a cohort of older controls (black). (TIF) [file pone.0036278.s001.tif]
