# Supplementary material for: Association of the CpG Methylation Pattern of the Proximal Insulin Gene Promoter with Type 1 Diabetes
Source: PLoS One. 2012 May 2;7(5):e36278. doi: 10.1371/journal.pone.0036278 (PMC3342174; doi:10.1371/journal.pone.0036278)
Supplement: Table S2 — Main characteristics of T1D patients and age-matched non diabetic controls. Results are expressed as mean ± sd. (DOCX) [file pone.0036278.s003.docx]

Table S2. Main characteristics of T1D patients and age-matched non diabetic controls. Results are expressed as mean ± sd.

|  |  |  |
| --- | --- | --- |
|  | **T2D PATIENTS** | **T2D CONTROLS** |
|  |  |  |
|  |  |  |
| **N** | *132* | *186* |
| **Sex (M/F)** | 101/31 | 105/81 |
| **Current age (yrs)** | 54.4 ± 4.5 | 52.4 ± 4.5 |
| **BMI (kg/m^2^)** | 29.8 ± 3.4 | 25.7 ± 3.3 |
| **Age at clinical onset (yrs)** | 48.5 ± 5.4 | - |
| **Diabetes Duration** | 5.9 ± 4.6 | - |
|  |  |  |
